# Association Between Health Literacy and Salt-Related Knowledge, Attitudes, and Practices: A Path Analysis of Indirect Associations via eHealth Literacy and Information Sources

**DOI:** 10.3390/nu18060916

**Published:** 2026-03-13

**Authors:** Naibo Wang, Yuanzhi Li, Chen Wang, Yuanan Lu, Dezhi Wan, Tian Lu, Lewei Xu, Xiong Liao, Lei Wu

**Affiliations:** 1 School of Public Health, Jiangxi Provincial Key Laboratory of Disease Prevention and Public Health, Jiangxi Medical College, Nanchang University, Nanchang 330006, China; 2 Jiangxi Provincial Center for Patriotic Health and Health Promotion, Nanchang 330003, China; 3 Environmental Health Laboratory, Department of Public Health Sciences, Thompson School of Social Work and Public Health, University of Hawaii at Manoa, Honolulu, HI 96822, USA; 4 Jiangxi Association for Health Education and Tobacco Control, Nanchang 330003, China; 5 Department of Nutrition, The First Affiliated Hospital, Jiangxi Medical College, Nanchang University, Nanchang 330006, China

**Keywords:** salt-related knowledge, attitudes, and practices, salt reduction, health literacy, eHealth literacy, path analysis

## Abstract

**Background**: Reducing dietary salt intake is a global public health priority. However, empirical evidence is needed to clarify whether higher levels of health literacy (HL) and eHealth literacy (eHL), together with the use of diversified information dissemination channels, are positively associated with better salt-related knowledge, attitudes, and practices (KAP). This study examined the indirect associations via eHL and the number of sources of salt-reduction information (NSSI) in the relationship between HL and salt-related KAP. **Methods**: A cross-sectional survey was conducted from 2022 to 2023 using multistage stratified random sampling among residents aged 15–69 in 22 counties/districts of Jiangxi Province, China. Data on sociodemographic characteristics, HL, eHL, NSSI, and salt-related KAP were collected through face-to-face household interviews using a standardized electronic questionnaire system. Spearman correlation analysis and multiple linear regression were applied to assess associations among HL, eHL, NSSI, and salt-related KAP. Path analysis was employed to evaluate the indirect associations between HL and salt-related KAP via eHL and NSSI. **Results**: A total of 5396 residents participated, of whom 51.50% were male. Participants aged 15–34, 35–54, and 55–69 years accounted for 13.10%, 42.96%, and 43.94% of the sample, respectively. After adjustment for covariates, individuals with adequate HL, adequate eHL, and a greater NSSI had significantly higher total salt-related KAP scores (*p* < 0.001). In the path analysis, the standardized direct association of HL with the total salt-related KAP was 0.229 (*p* < 0.001). The standardized indirect associations via NSSI and eHL were 0.089 (95% CI: 0.069 to 0.111, *p* < 0.001) and 0.057 (95% CI: 0.033 to 0.089, *p* < 0.001), respectively, accounting for 23.73% and 15.20% of the total association. **Conclusions**: High levels of HL and eHL, together with increased exposure to multiple salt-reduction information sources, are associated with improved salt-related KAP. Both eHL and NSSI partially explain the association between HL and salt-related KAP. Future salt-reduction interventions should integrate conventional health education with mobile health technologies to expand information dissemination channels and support sustained salt-reduction behaviors.

## 1. Introduction

Non-communicable diseases (NCDs) have become the leading threat to global health, with cardiovascular diseases (CVDs) accounting for the greatest proportion of morbidity and mortality worldwide [1]. Hypertension, a major modifiable risk factor for CVDs, continues to increase in prevalence, posing sustained challenges to public health systems and economic development. Substantial epidemiological evidence demonstrates that excessive sodium intake is a primary contributor to elevated blood pressure [2,3]. Beyond hypertension, high salt consumption has been linked to an increased risk of gastric cancer, obesity, chronic kidney disease, osteoporosis, and cerebral small vessel disease [4,5,6,7]. It is estimated that approximately 1.9 million deaths globally each year are attributable to excessive salt intake [3]. Consequently, the World Health Organization (WHO) has identified population-wide salt reduction as one of the most cost-effective strategies for the prevention of NCDs.

Dietary behaviors are strongly influenced by individuals’ knowledge and attitudes toward nutrition. Individuals with higher levels of dietary knowledge and positive health attitudes are more likely to engage in healthy eating behaviors [8,9]. Studies applying the Knowledge–Attitudes–Practices (KAP) framework to salt consumption have consistently shown that attitudes are positively associated with salt-reduction behaviors, while knowledge exerts a direct influence on both attitudes and practices [10,11]. In contrast, individuals holding negative attitudes toward salt reduction are less likely to reduce sodium intake [12]. These findings underscore the importance of improving salt-related knowledge and attitudes as key pathways for promoting salt-reduction behaviors.

In recent years, health literacy (HL), defined as the ability to access, understand, appraise, and apply health information for informed decision-making, has received increased attention in public health research [13]. Individuals with higher HL are better able to recognize the health risks associated with excessive salt intake and are more likely to translate health knowledge and positive attitudes into appropriate behaviors. With the rapid extension of digital health information, eHealth literacy (eHL) has emerged as an extension of HL in digital contexts, emphasizing the capacity to seek, evaluate, and use health information from electronic sources, including web-based and mobile platforms [14]. Higher eHL has been consistently associated with more proactive and health-promoting behaviors. For example, during periods characterized by substantial information overload, such as the COVID-19 pandemic, higher eHL enabled individuals to more effectively filter misinformation and adopt protective health behaviors [15]. A recent meta-analysis has further confirmed a stable positive association between eHL and a wide range of health behaviors, highlighting its important facilitating and explanatory role [16]. In addition to individual literacy levels, the channels through which health information is disseminated play a critical role in shaping dietary behaviors. Evidence from salt-reduction interventions suggests that exposure to multiple information sources can enhance public awareness and promote behavioral change. For instance, a national consumer awareness campaign in Australia demonstrated that digital media promotion improved public knowledge of salt reduction and contributed to healthier dietary behaviors [17]. Similarly, a systematic review reported that diversified communication strategies, including health education programs and media campaigns, effectively increased awareness of the health risks of high salt intake and facilitated favorable changes in attitudes and practices [18]. Collectively, these findings suggest that the association of HL with salt-related KAP may operate through multiple pathways—specifically, by improving individuals’ ability to access and use salt-reduction information and by enhancing eHL, which enables more effective utilization of digital health resources. Theoretically, these interrelationships can be conceptualized through the Integrated Model of Health Literacy [19]. As a comprehensive framework, this model asserts that health-promoting outcomes are primarily determined by the continuous process of accessing, understanding, appraising, and applying health information. Within the context of salt reduction, HL serves as the capacity that catalyzes this process. Higher HL is associated with increased proactive exposure to varied salt-reduction information channels and corresponds to advanced eHL. Consequently, enhanced information access and advanced digital skills collectively facilitate improvements in salt-related knowledge, attitudes and practices.

Despite growing recognition of the importance of HL, eHL, and information dissemination in salt reduction, empirical studies examining their interrelationships within a unified analytical framework remain limited. In particular, there is a lack of evidence assessing the indirect pathways involving eHL and exposure to multiple salt-reduction information sources in the association between HL and salt-related KAP. Therefore, this study investigated residents aged 15–69 years in 22 districts/counties of Jiangxi Province, China, to examine the associations among HL, eHL, NSSI, and salt-related KAP. Furthermore, we employed path analysis to assess the indirect associations via eHL and NSSI in the relationship between HL and salt-related KAP. The findings of this study aim to provide empirical evidence to inform the development of targeted, evidence-based, and sustainable salt-reduction interventions that integrate health education with digital health strategies.

## 2. Materials and Methods

### 2.1. Study Design and Participants

A cross-sectional survey was conducted during 2022 and 2023 across 22 counties/districts in Jiangxi Province, China. To obtain a representative sample, a multistage stratified sampling design was used, combining probability proportional to size (PPS) sampling with simple random sampling. Population data were obtained from the Seventh National Population Census. To account for household clustering, only one eligible participant was selected from each household. The sampling procedure consisted of five stages: (1) Twenty-two counties/districts were selected from all counties in Jiangxi Province using PPS sampling. (2) Three subdistricts/townships were selected within each sampled county/district using PPS sampling. (3) Two communities/villages were randomly selected from each sampled subdistrict/township using PPS sampling. (4) Forty households were randomly selected from each community/village using simple random sampling. (5) One household member was randomly selected using the Kish grid method.

Eligible participants were residents aged 15–69 years who were not living in collective dormitories. Inclusion criteria included (1) residence in the local area for more than 6 months cumulatively during the previous 12 months; (2) age between 15 and 69 years; and (3) willingness to participate. Exclusion criteria were: (1) residence in collective settings such as military bases, prisons, or nursing homes; and (2) inability or unwillingness to participate due to cognitive or physical limitations.

### 2.2. Sample Size Estimation

Based on previous studies, the mean salt-related Knowledge, Attitudes, and Practices (KAP) score in China was estimated at 31.27 with a standard deviation (σ) of 9.18 [20]. A two-sided significance level (α) of 0.05, a relative error of 3%, and a design effect of 4.0 were assumed to account for the multistage sampling design. After allowing for an anticipated non-response rate of 15%, the minimum required sample size was estimated to be 3462. The final sample included 5396 participants from 22 counties, exceeding this requirement. In addition, a Monte Carlo simulation was conducted to estimate the sample size required for the parallel mediation model. Assuming a correlation coefficient of 0.1 among the independent, mediating, and dependent variables, a simulation based on 20,000 draws and 1000 replications indicated that a sample size of 5000 would provide statistical power greater than 0.90 to detect the expected mediation effects.

### 2.3. Data Collection and Quality Control

Before data collection, a three-level coordination structure (provincial, municipal, and county levels) was established to oversee organization, technical support, and implementation. Investigators and quality control staff were recruited from health promotion centers, centers for disease control and prevention, township health centers, and community health service centers. All personnel received standardized training on survey procedures and quality control protocols.

Household sampling was conducted using resident lists from selected communities or villages, followed by face-to-face household interviews. Interviewers explained the study purpose, ensured confidentiality, and obtained informed consent before data collection. Data were collected using the Health Literacy Survey App (v4.0.1) developed by Nanjing Weiynet Technology Co., Ltd. (Nanjing, China). The standardized electronic questionnaire system enabled real-time data entry and automated logic checks. Interviewers recorded responses on-site and were prohibited from altering questions or skipping items. As all questionnaire items were mandatory, there were no item-level missing data among the participants who completed the entire assessment. However, 69 individuals (1.28%) did not complete the health literacy assessment. These participants were retained in the final analysis as they provided complete data for all other variables. Quality control staff reviewed data validity and integrity through backend monitoring, including audio verification and field inspections.

### 2.4. Measurements

#### 2.4.1. Basic Characteristics

Sociodemographic covariates included age, gender, education level, county/district, smoking status, and physical activity. Smoking was defined as having smoked within the past month, excluding former smokers. Physical activity was defined as engaging in moderate-intensity or higher exercise at least three times per week for ≥30 min per session.

#### 2.4.2. Health Literacy (HL)

HL was assessed using a standardized questionnaire developed by the Chinese Center for Health Education [21]. The instrument measures the ability to access, understand, and apply health information and has demonstrated good reliability and validity in national HL. In this study, the Cronbach’s α for the HL questionnaire was 0.914. It covers three domains: basic health knowledge and beliefs, healthy lifestyles and behaviors, and basic skills. Total scores range from 0 to 66. To align with the official, standardized criteria defined by the Chinese National Health Literacy Surveillance guidelines, a score of ≥53 (80%) was used to indicate adequate HL.

#### 2.4.3. eHealth Literacy (eHL)

eHL was assessed using the Chinese version of the eHealth Literacy Scale, developed by Norman et al. [22]. In this study, the scale demonstrated good internal consistency, with a Cronbach’s α of 0.971. The scale includes eight items evaluating the ability to seek, appraise, and apply electronic health information, rated on a 5-point Likert scale (1 = Strongly disagree to 5 = Strongly agree). Total scores range from 8 to 40. To maintain consistency with the national HL standards and previous studies [22,23], a score of ≥32 (80%) was used to indicate adequate eHL.

#### 2.4.4. Number of Sources of Salt-Reduction Information (NSSI)

Participants reported salt-reduction information sources through a two-step process. Those who indicated prior exposure selected applicable sources from a list of 10 options: healthcare providers, family, friends, community channels, print media, television, radio, internet/social media, public transportation media, and others. Based on response distribution and gradients of information exposure, NSSI was categorized as 0, 1–2, 3–4, 5–6, and ≥7 sources.

#### 2.4.5. Salt-Related KAP

The salt-related KAP questionnaire was adapted from previous studies and validated through a pilot study [20,24]. The instrument consists of three dimensions (knowledge, attitudes, and practices), with two to six items per dimension. Consistent with previous research [24], to standardize the scoring across varying question formats, each item was converted to a 0 to 10-point scale. For frequency-based behavioral items, a proportional scoring system was applied to reflect the gradient of salt-related practices. The infrequent “don’t know” responses in salt-related practices were handled using mode imputation. Dimension scores were calculated as the mean item scores (range: 0–10), and the total KAP score ranged from 0 to 30, with higher scores indicating greater salt-related awareness and healthier practices related to salt. Finally, the instrument demonstrated good construct validity (CFI and TLI > 0.9) and internal consistency (Cronbach’s α > 0.75) overall and across all dimensions. Details of the questionnaire and scoring criteria are provided in Table S1.

### 2.5. Statistical Analysis

Continuous variables were summarized as mean (SD), and categorical variables as *n* (%). Differences in KAP scores were assessed using *t*-tests or ANOVA. Covariates evaluated in this study included age, gender, county/district, education, smoking, and physical activity. Spearman and partial correlation analyses (adjusting for covariates) examined associations among HL, eHL, NSSI, and salt-related KAP. Multiple linear regression models were constructed to analyze the associations of HL, eHL and NSSI with KAP scores, comprising both an unadjusted model (Model 1) and a model adjusted for covariates (Model 2).

Four path models (Model K, Model A, Model P, and Model T) were constructed. In these models, HL served as the predictor variable; NSSI and eHL served as parallel intermediate variables; and salt-related knowledge, attitudes, behaviors, and total KAP score served as the respective outcome variables. In all models, every estimated pathway was consistently adjusted for the aforementioned covariates. WLSMV estimation was used, with HL, eHL, and NSSI specified as categorical variables to ensure robustness. Missing data for HL, resulting from 69 participants (1.28%) failing to complete this specific module, were handled using pairwise deletion. Standardized path coefficients were reported to facilitate the comparison of the strength of associations across different pathways. The significance of the indirect associations was tested using the Bootstrap method with 5000 resamples. To account for the clustering effect in the multistage sampling design, multiple linear regression and path models were adjusted for clustering at the subdistrict/township level. Analyses were performed using SAS 9.4 and Mplus 8.3, with *p* < 0.05 considered statistically significant.

## 3. Results

### 3.1. Basic Characteristics and Salt-Related KAP

A total of 5396 residents participated in the survey, of whom 69 (1.28%) did not complete the HL assessment but provided responses to all other survey questions. Most participants were aged 55–69 years (43.94%) and 35–54 years (42.96%), while those aged 15–34 years accounted for 13.10%. Males comprised 51.50% of the sample. Regarding salt-reduction information acquisition, 42.14% of participants accessed 1–2 information sources, whereas 31.62% reported receiving no salt-related information, with no participants selecting the ‘others’ option. The proportions of participants with adequate HL and eHL were 21.36% and 20.46%, respectively, indicating an overall low level.

Significant differences in salt-related KAP scores were observed across subgroups. Participants aged 35–54 years showed the highest attitudes, practices, and total KAP scores. Females scored significantly higher than males across these metrics, although their knowledge scores were slightly lower. Additionally, higher education, non-smoking status, and regular physical exercise were associated with higher total KAP scores. Participants with more information sources (NSSI), as well as those with adequate HL and eHL, demonstrated significantly higher KAP scores (*p* < 0.001). Details are shown in Table 1, and item-level responses are presented in Table S2.

### 3.2. Associations of HL, eHL, and NSSI with Salt-Related KAP

**Figure 1:**
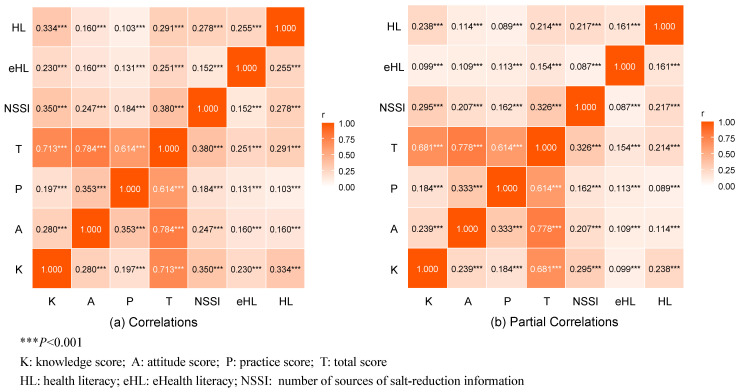
Correlations and partial correlations among HL, eHL, NSSI, and salt-related KAP.

As shown in Figure 1, all variables were positively correlated (*p* < 0.001). Knowledge was significantly correlated with attitudes (*r* = 0.280) and practices (*r* = 0.197), with the strongest correlation observed between attitudes and practices (*r* = 0.353). Total KAP showed the strongest association with NSSI (*r* = 0.380), while HL was most strongly associated with salt-related knowledge (*r* = 0.334). These associations remained significant after covariate adjustment, although correlation coefficients were slightly attenuated.

Multiple linear regression analyses (Table 2) indicated that HL, eHL, and NSSI were significant predictors of salt-related KAP. In the adjusted model (model 2), total KAP scores increased significantly with higher NSSI; participants accessing 7–9 sources had the largest increase compared with those with none (*β* = 4.050, 95% CI: 3.217 to 4.883, *p* < 0.001). Adequate HL (*β* = 1.782, 95% CI: 1.370 to 2.194) and adequate eHL (*β* = 1.409, 95% CI: 0.958 to 1.859) were also associated with higher total KAP scores (*p* < 0.001). Similar positive associations were observed for knowledge, attitudes, and practices (Tables S3–S5).

### 3.3. Path Analysis

**Figure 2:**
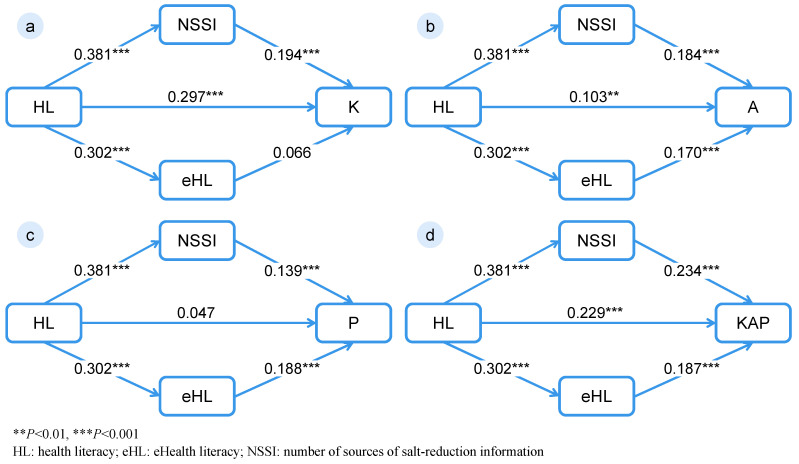
Path models illustrating the standardized indirect associations of HL with salt-related knowledge (**a**), attitudes (**b**), practices (**c**), and total KAP score (**d**) via eHL and NSSI.

Four path models were constructed with salt-related Knowledge (Model K), Attitudes (Model A), Practices (Model P), and Total KAP Score (Model T) as outcome variables. All models showed excellent goodness of fit (χ^2^/df = 4.552–4.556, TLI > 0.94, CFI > 0.99, RMSEA = 0.026, and SRMR = 0.005, Table 3). All path coefficients were statistically significant, except for the direct associations of HL with salt-related practices and eHL with salt-related knowledge. Across KAP dimensions, the indirect pathway via NSSI showed the strongest magnitude for salt-related knowledge, while eHL accounted for the largest proportion of the total association with salt-related practices (Figure 2 and Tables S6–S8).

As shown in Figure 2 and Table 4, in the total KAP model (Model T), HL had a significant standardized direct association with salt-related KAP (*β* = 0.229, *p* < 0.001). Both NSSI and eHL acted as significant parallel intermediate variables. The standardized estimates for the indirect associations via NSSI and eHL were 0.089 (95% CI: 0.069 to 0.111) and 0.057 (95% CI: 0.033 to 0.089), accounting for 23.73% and 15.20% of the total association, respectively.

## 4. Discussion

Based on a large-scale cross-sectional survey, this study examined the associations among health literacy (HL), eHealth literacy (eHL), the number of sources of salt-reduction information (NSSI), and salt-related knowledge, attitudes, and practices (KAP). The findings revealed that both eHL and NSSI are independently associated with salt-related KAP and, importantly, function as significant intermediate variables in the relationship of HL with salt-related practices. These results provide empirical evidence supporting a multi-pathway framework through which HL is linked to dietary behaviors related to salt intake.

Consistent with previous studies, overall awareness of the health risks associated with excessive salt intake was relatively high among participants; however, knowledge of specific recommendations—such as the recommended daily intake and interpretation of salt-related nutrient labeling remained limited, with the salt-related practices score being the lowest among the three dimensions. This discrepancy highlights a persistent “knowledge-practice gap”, which has been widely reported in salt-related KAP research [20,23,25]. Sociodemographic disparities were also evident. Participants with higher education attainment, urban residence, and females exhibited significantly higher salt-related KAP scores, likely reflecting greater access to health information and stronger capacity to process and apply nutritional guidance [12,26,27]. In addition, non-smokers and individuals engaging in regular physical activity demonstrated more favorable salt-related KAP, suggesting a clustering or synergistic pattern of health-promoting behaviors. Such synergy may be partially explained by shared psychosocial mechanisms, including perceived health benefits, self-efficacy, and health consciousness, which collectively facilitate the translation of knowledge into healthy practices [28,29]. These findings underscore the importance of prioritizing vulnerable populations—particularly individuals with lower education levels, rural residents, and those with unhealthy lifestyle behaviors—when designing targeted salt-reduction interventions.

A key contribution of this study lies in elucidating the pathways through which HL is associated with salt-related KAP. While HL showed a significant direct association with salt-related knowledge, its direct association with salt-related practices was relatively weak and not statistically significant (*β* = 0.047, *p* = 0.152), indicating that behavior change is largely explained by other factors. Both NSSI and eHL demonstrated significant indirect associations, supporting the hypothesis that HL relates to behavior indirectly through enhanced access to information and improved capacity to process and apply health information. Specifically, NSSI consistently served as an indirect pathway in the association between HL and all dimensions of salt-related KAP, suggesting that individuals with higher HL are more likely to access multiple sources of salt-reduction information, thereby reinforcing knowledge acquisition and favorable attitudes and behaviors [30,31,32]. Notably, although the indirect association via eHL with knowledge was not significant, the pathway via eHL accounted for a substantial proportion (36.31%) of the total association with salt-related practices (*p* < 0.001), exceeding that of NSSI. This finding aligns with previous research [33] and highlights the critical role of eHL in translating information into action in contemporary digital environments. By enabling individuals to actively seek, evaluate, and internalize health information, eHL appears to play a particularly important role in facilitating behavioral change [34]. Beyond merely improving access to digital platforms, eHL equips individuals with the specific skills required to appraise and apply health information. Individuals with advanced eHL are better positioned to filter out dietary misinformation, correctly interpret digital nutrition labels, and select evidence-based strategies. Consequently, these specific pathways enable them to translate online information into tangible daily practices, such as modifying dietary habits and purchasing low-sodium alternatives. Taken together, NSSI and eHL operate as intermediate variables, linking HL to behavioral outcomes through complementary mechanisms—namely, increased opportunities for information exposure and enhanced capacity for information filtering and application.

The present findings suggest that expanding dissemination channels and strengthening eHL are effective strategies for improving salt-related KAP at the population level. With rapid advances in digital technology, new media platforms and mobile health (mHealth) tools have become increasingly influential in health communication and behavioral interventions. Compared with traditional health education approaches, mHealth technologies offer several advantages, including real-time feedback, personalized content delivery, and interactive engagement, which can enhance both reach and effectiveness. Evidence from intervention studies supports these benefits. For example, integrating digital tools, such as food-label scanning applications, targeted text messaging, and interactive tracking platforms, has been shown to effectively promote low-salt food choices and overall adherence to healthy behaviors [35,36]. Furthermore, incorporating real-time monitoring and social interaction features into these platforms can significantly reduce isolation, strengthen social support, and increase motivation for sustained behavior change across diverse populations [37,38]. Extending mHealth interventions to school and community settings has further amplified their impact, underscoring their potential for multi-level and population-wide implementation [39,40]. Collectively, these findings support the development of integrated intervention models that combine broad-coverage traditional health education with behavior-oriented digital technology to achieve sustainable salt reduction.

Several limitations of this study should be acknowledged. First, the cross-sectional design precludes causal inference regarding the observed associations [41,42]. Specifically, this design cannot confirm that HL directly drives better KAP through eHL and NSSI. Instead, it is plausible that individuals interested in healthy behaviors may seek more information, which in turn fosters higher literacy. Second, while the mandatory response design of our electronic system effectively prevented item omission, the reliance on self-reported data obtained through questionnaires and face-to-face interviews may introduce recall and social desirability biases. Third, as the study was conducted in Jiangxi Province, a less economically developed region in China with specific dietary cultures, including a traditional preference for high-salt and pickled foods, the salt-related KAP and its associations with HL observed in this study may differ from those in other regions. Therefore, the generalizability of our findings to broader contexts or populations may be limited. In addition, although operationalizing information exposure as a quantitative count (NSSI) effectively captures the breadth of information access, it may not fully reflect the qualitative disparities in reliability among channels—such as the difference between professional healthcare guidance and unverified social media content.

While this study focused on individual HL, salt consumption is profoundly shaped by broader structural and contextual determinants. Factors such as cultural dietary patterns, the modern food environment, food industry practices (e.g., hidden sodium in ultra-processed foods), and socioeconomic status often create significant barriers to salt reduction, even for individuals with high HL. Therefore, comprehensive strategies must complement individual-level education with macro-level environmental and policy interventions. Future research should incorporate longitudinal or experimental designs, include objective measures of salt intake such as 24 h urinary sodium excretion, evaluate the qualitative differences among different health information sources, and explore additional psychosocial factors—such as social support and individual motivation—to further elucidate the mechanisms underlying salt-related behavior change.

In summary, this study demonstrates that HL, eHL, and diversified sources of salt-reduction information are jointly associated with improved salt-related KAP. The synergistic interplay among these factors provides a robust theoretical and empirical foundation for comprehensive salt reduction strategies. Policymakers and public health practitioners should adopt multifaceted approaches that expand communication channels while simultaneously enhancing population-level HL and eHL. By leveraging digital health technologies and fostering cross-sectoral collaboration, it is possible to establish an integrated, multi-setting intervention system with broad population coverage. Such efforts are essential for promoting the adoption and maintenance of salt-reduction behaviors and for achieving long-term population-level reduction in dietary salt intake.

## 5. Conclusions

Salt-related knowledge, attitudes, and practices are jointly associated with sociodemographic characteristics, health literacy (HL), eHealth literacy (eHL), and the number of sources of salt-reduction information (NSSI). The present findings demonstrate that eHL and NSSI function as significant intermediate factors in the association between HL and salt-related KAP, representing key indirect pathways linking health literacy to salt-reduction practices. These results underscore the importance of both access to credible health information and the capacity to critically evaluate and apply such information in supporting favorable dietary behaviors. Accordingly, future salt-reduction strategies should adopt integrated approaches that combine conventional health education with mobile health (mHealth)-based interventions, expand and diversify information dissemination channels, and systematically strengthen population-level HL and eHL. Such evidence-informed strategies are likely to enhance the effectiveness and sustainability of salt-reduction interventions and contribute to achieving population-level reduction in dietary salt intake.

## Figures and Tables

**Table 1:** Baseline characteristics and salt-related KAP.

		Participants	Knowledge Score		Attitudes Score		Practices Score		Total Score	
		*n* (%)	Mean (SD)	*p*	Mean (SD)	*p*	Mean (SD)	*p*	Mean (SD)	*p*
Age group										
	15–34	707 (13.10)	6.28 (2.26)	<0.001	7.78 (2.72)	<0.001	5.75 (1.81)	<0.001	19.81 (4.79)	<0.001
	35–54	2318 (42.96)	5.87 (2.49)		7.85 (2.92)		6.42 (1.74)		20.13 (5.24)	
	55–69	2371 (43.94)	4.93 (2.53)		7.27 (3.24)		6.26 (1.64)		18.47 (5.50)	
Gender										
	Male	2779 (51.50)	5.61 (2.47)	0.003	7.42 (3.13)	<0.001	6.04 (1.76)	<0.001	19.06 (5.39)	<0.001
	Female	2617 (48.50)	5.40 (2.60)		7.76 (2.96)		6.50 (1.64)		19.67 (5.31)	
Area										
	District	2717 (50.35)	5.78 (2.52)	<0.001	7.64 (3.03)	0.175	6.22 (1.80)	0.073	19.64 (5.45)	<0.001
	County	2679 (49.65)	5.24 (2.51)		7.53 (3.08)		6.30 (1.64)		19.07 (5.25)	
Education										
	Illiterate	449 (8.32)	3.42 (2.18)	<0.001	6.48 (3.59)	<0.001	6.01 (1.39)	<0.001	15.91 (5.15)	<0.001
	Primary school	1596 (29.58)	4.71 (2.42)		7.10 (3.24)		6.11 (1.62)		17.92 (5.32)	
	Middle school	1984 (36.77)	5.77 (2.40)		7.78 (2.98)		6.35 (1.76)		19.89 (5.19)	
	High school	805 (14.92)	6.59 (2.24)		8.16 (2.59)		6.45 (1.86)		21.21 (4.77)	
	College or above	562 (10.42)	7.01 (2.09)		8.34 (2.39)		6.32 (1.82)		21.67 (4.47)	
Smoking										
	Yes	1401 (25.96)	5.44 (2.47)	0.236	7.20 (3.23)	<0.001	5.93 (1.80)	<0.001	18.57 (5.48)	<0.001
	No	3995 (74.04)	5.54 (2.56)		7.72 (2.98)		6.38 (1.68)		19.63 (5.29)	
Physical activity										
	Yes	981 (18.18)	5.90 (2.52)	<0.001	8.08 (2.86)	<0.001	6.57 (1.73)	<0.001	20.55 (5.37)	<0.001
	No	4415 (81.82)	5.43 (2.53)		7.48 (3.08)		6.19 (1.71)		19.09 (5.32)	
NSSI										
	0	1706 (31.62)	4.40 (2.37)	<0.001	6.53 (3.36)	<0.001	5.83 (1.53)	<0.001	16.76 (4.94)	<0.001
	1–2	2274 (42.14)	5.60 (2.46)		7.83 (2.94)		6.35 (1.79)		19.78 (5.28)	
	3–4	564 (10.45)	6.44 (2.32)		8.15 (2.70)		6.50 (1.71)		21.09 (5.00)	
	5–6	511 (9.47)	6.65 (2.21)		8.66 (2.21)		6.67 (1.67)		21.98 (4.22)	
	7–9	341 (6.32)	7.23 (2.07)		8.70 (2.17)		6.83 (1.76)		22.76 (4.11)	
eHL										
	Adequate	1104 (20.46)	6.63 (2.24)	<0.001	8.55 (2.39)	<0.001	6.72 (1.80)	<0.001	21.90 (4.59)	<0.001
	Inadequate	4292 (79.54)	5.22 (2.53)		7.34 (3.15)		6.14 (1.68)		18.70 (5.35)	
HL *										
	Adequate	1138 (21.36)	7.09 (1.99)	<0.001	8.54 (2.40)	<0.001	6.59 (1.76)	<0.001	22.22 (4.28)	<0.001
	Inadequate	4189 (78.64)	5.05 (2.49)		7.34 (3.16)		6.17 (1.69)		18.56 (5.36)	

* 69 participants did not complete the health literacy assessment. NSSI: Number of Sources of Salt-Reduction Information; eHL: eHealth Literacy; HL: Health Literacy.

**Table 2:** Associations of HL, eHL, and NSSI with the total salt-related KAP score.

		Model 1			Model 2		
		*β*	95% CI	*p*	*β*	95% CI	*p*
NSSI							
	0	Ref	-	-	Ref	-	-
	1–2	2.677	2.184 to 3.170	<0.001	2.407	1.930 to 2.885	<0.001
	3–4	3.785	2.908 to 4.662	<0.001	3.229	2.396 to 4.061	<0.001
	5–6	4.293	3.568 to 5.018	<0.001	3.736	3.061 to 4.410	<0.001
	7–9	4.706	3.858 to 5.555	<0.001	4.050	3.217 to 4.883	<0.001
eHL							
	Adequate	1.963	1.520 to 2.407	<0.001	1.409	0.958 to 1.859	<0.001
	Inadequate	Ref	-	-	-	-	-
HL							
	Adequate	2.117	1.689 to 2.546	<0.001	1.782	1.370 to 2.194	<0.001
	Inadequate	Ref	-	-	-	-	-

NSSI: number of sources of salt-reduction information; eHL: eHealth literacy; HL: health literacy. Ref: reference group. -: not applicable. Model 1 included HL, eHL, and NSSI. Model 2 further adjusted for age, gender, county/district, education, smoking, and physical activity.

**Table 3:** Fit indices for the path models of salt-related KAP.

	χ^2^/df	CFI	TLI	RMSEA (90% CI)	SRMR
Model K	4.553	0.999	0.958	0.026 (0.006 to 0.051)	0.005
Model A	4.552	0.998	0.942	0.026 (0.006 to 0.051)	0.005
Model P	4.552	0.998	0.944	0.026 (0.006 to 0.051)	0.005
Model T	4.556	0.999	0.960	0.026 (0.006 to 0.051)	0.005

Model K, Model A, Model P, and Model T represent the path models for salt-related knowledge, attitude, practice, and total KAP score, respectively.

**Table 4:** Standardized results of the path model for the total salt-related KAP score.

	Estimate	SE	*p*	95% CI	Proportion * (%)
Indirect association 1	0.089	0.011	<0.001	0.069 to 0.111	23.73
Indirect association 2	0.057	0.014	<0.001	0.033 to 0.089	15.20
Direct association	0.229	0.038	<0.001	0.154 to 0.302	61.07
Total association	0.375	0.032	<0.001	0.309 to 0.436	-

Indirect association 1: HL → NSSI → Salt-related KAP. Indirect association 2: HL → eHL → Salt-related KAP. * Refers to the ratio of the specific association to the total association.

## Data Availability

The data used or analyzed in this study are available from the corresponding author on reasonable request.

## Supplementary Materials

The following supporting information can be downloaded at: https://www.mdpi.com/article/10.3390/nu18060916/s1, Table S1: Scoring criteria for salt related knowledge, attitudes and practices; Table S2: Responses to individual items of the salt reduction KAP; Table S3: Associations of HL, eHL, and NSSI with the salt-related knowledge score; Table S4: Associations of HL, eHL, and NSSI with the salt-related attitudes score; Table S5: Associations of HL, eHL, and NSSI with the salt-related practices score; Table S6: Standardized results of the path model for the salt-related knowledge score; Table S7: Standardized results of the path model for the salt-related attitudes score; Table S8: Standardized results of the path model for the salt-related practices score.
