# Supplementary material for: Association Between Health Literacy and Salt-Related Knowledge, Attitudes, and Practices: A Path Analysis of Indirect Associations via eHealth Literacy and Information Sources
Source: Nutrients. 2026 Mar 13;18(6):916. doi: 10.3390/nu18060916 (PMC13029024; doi:10.3390/nu18060916)
Supplement: Supplementary file 1 [file nutrients-18-00916-s001.zip › nutrients-4173530-supplementary.pdf]

Table S1. Scoring criteria for salt related knowledge, attitudes and practices.

Questions	Scoring method
**Knowledge**	
**K1.How many grams of salt should be consumed for healthy adults per day?**	option 1 = 10 points; else = 0 points.
1=≤5g	
2=≤8g	
3=≤10g	
999=don't know	
**K2.What problems can result from consuming too much salt over a long period of time?**	option 1 = 10 points; else = 0 points.
1=Elevated blood pressure	
2=Reduced blood pressure	
3=No impact	
999=Don't Know	
**K3.Which item in the food nutrition label indicates the salt content?**	option 5 = 10 points; else = 0 points.
1=Energy	
2=Protein	
3=Fat	
4=Carbohydrates	
5=Sodium	
999=Don't Know	
**K4.Does eating less salt lead to the lack of strength?**	option 2 = 10 points; else = 0 points.
1=Yes	
2=No	
999=Don't Know	
**K5.Does eating less salt cause hair to turn white?**	option 2 = 10 points; else = 0 points.
1=Yes	

2=No
999=Don't Know

K6.Which of the following foods or condiments have a higher salt content?(Please select all that apply)

Each option earns 1 point

fine dried noodles/instant noodles/ham sausage/sauced beef/soy sauce/monosodium glutamate/spiced melon seeds/ preserved plum/soda biscuits/rice crust

Attitudes

A1.What would you think if food were cooked using less salt at home?

option 2 = 10 points; option 3 = 5 points; option 1 = 0 points.

1=Less salt means it's not delicious
2=Less salt is good for health
3=Not very concerned about saltiness

A2.Are you willing to choose less salty diet?

option 1 = 10 points; option 2 = 5 points; option 3 = 0 points.

1=Willing and believing that I can do it
2=Willing, but not confident that I can do it
3=Unwilling

Practices

P1.What is your usual taste when eating?

option 3 = 10 points; option 2 = 5 points; option 1 = 0 points.

1=More Salty
2=Moderate
3=Less Salty

P2.How many times do you usually eat pickled food (such as salted vegetables, pickles, salted eggs, and sauced meat) in a week?

option 4 = 10 points; option 3 = 6.6 points; option 2 = 3.3 points;option 1 = 0 points.
option 999 was calculated as option 4 (Mode).

1=Almost every day
2=3-5 days
3=1-2 days
4=No more than once
999=Don't Know

P3.How often have you eaten out or ordered takeout over the past month?

option 4 = 10 points; option 3 = 6.6 points; option 2 = 3.3 points;option 1 = 0 points.

1=Almost every day
2=4 or more times per week
3=1-3 times per week
4=Almost none

P4.Do you proactively request less salt when dining out or ordering takeout?†

option 5 = 10 points; option 4 = 7.5 points; option 3 = 5 points; option 2 = 2.5 points; option 1 = 0 points.

1=Never
2=Occasionally (1-2 times out of 10)
3=Sometimes (3-5 times out of 10)
4=Frequently (6-9 times out of 10)
5=Every time

P5.Which of the following methods have you used to reduce salt when dining out or ordering takeout?† (Please select all that apply)

Each choice earns 2.5 point

1=Choose dishes that are steamed, boiled, braised and mixed
2=Try not to order salted, pickled, and marinated products
3=Rinse food with heavy oil and salt before eating
4=Not or try not to drink salty leftover soup

P6.Do you currently use low-sodium salt in your home?

option 1 = 10 points; option 2 or 3 = 5 points; option 4 = 0 points.(option 2 or 3 indicates knowing what

	low-sodium salt is)
1=Yes<br>2=No<br>3=Don’t know<br>4=Haven’t heard of low-sodium salt	

†Those who have never eaten out or ordered takeout in the past month were not included.

Score of knowledge = (K1+K2+K3+K4+K5+K6)/6. Score of attitudes = (A1+A2)/2. Score of practices= (P1+P2+P3+P4+P5+P6)/(4+n); If the choice for P3 was 'Almost never' (both P4 and P5 have no answers), then n = 0; otherwise, n = 2. Total score = score of knowledge + score of attitudes+ score of practices.

Table S2. Responses to individual items of the salt reduction KAP

Questions	n	%
**Knowledge**		
**K1.How many grams of salt should be consumed for healthy adults per day?**		
≤5g	2618	48.52
≤8g	1012	18.75
≤10g	414	7.67
Don't know	1352	25.06
**K2.What problems can result from consuming too much salt over a long period of time?**		
Elevated blood pressure	4366	80.91
Reduced blood pressure	140	2.59
No impact	245	4.54
Don't Know	645	11.96
**K3.Which item in the food nutrition label indicates the salt content?**		

Energy	263	4.87
Protein	199	3.69
Fat	117	2.17
Carbohydrates	536	9.93
Sodium	2586	47.92
Don't Know	1695	31.41
**K4.Does eating less salt lead to the lack of strength?**		
Yes	2428	45.00
No	2228	41.29
Don't Know	740	13.71
**K5.Does eating less salt cause hair to turn white?**		
Yes	744	13.79
No	3082	57.12
Don't Know	1570	29.09
**K6.Which of the following foods or condiments have a higher salt content?(Please select all that apply) mean (SD)**		
fine dried noodles/instant noodles/ham sausage/sauced beef/soy sauce/monosodium glutamate/spiced melon seeds/ preserved plum/soda biscuits/rice crust		5.49 (2.67)
**Attitudes**		
**A1.What would you think if food were cooked using less salt at home?**		
Less salt means it's not delicious	1103	20.44
Less salt is good for health	3700	68.57
Not very concerned about saltiness	593	10.99
**A2.Are you willing to choose less salty diet?**		

Willing and believing that I can do it	3465	64.21
Willing, but not confident that I can do it	1450	26.87
Unwilling	481	8.91
**Practices**		
**P1.What is your usual taste when eating?**		
More Salty	811	15.03
Moderate	3456	64.05
Less Salty	1129	20.92
**P2.How many times do you usually eat pickled food (such as salted vegetables, pickles, salted eggs, and sauced meat) in a week?**		
Almost every day	168	3.11
3-5 days	339	6.28
1-2 days	1067	19.77
No more than once	3638	67.42
Don't Know	184	3.41
**P3.How often have you eaten out or ordered takeout over the past month?**		
Almost every day	101	1.87
4 or more times per week	297	5.50
1-3 times per week	1018	18.87
Almost none	3980	73.76
***P4.Do you proactively request less salt when dining out or ordering takeout?**		
Never	835	58.97
Occasionally (1-2 times out of 10)	300	21.19
Sometimes (3-5 times out of 10)	141	9.96

Frequently (6-9 times out of 10)	68	4.80
Every time	72	5.08
***P5.Which of the following methods have you used to reduce salt when dining out or ordering takeout? (Please select all that apply)**		
Choose dishes that are steamed, boiled, braised and mixed		
Yes	526	37.15
No	890	62.85
Try not to order salted, pickled, and marinated products		
Yes	728	51.41
No	688	48.59
Rinse food with heavy oil and salt before eating		
Yes	260	18.36
No	1156	81.64
Not or try not to drink salty leftover soup		
Yes	287	20.27
No	1129	79.73
**P6.Do you currently use low-sodium salt in your home?**		
Yes	1103	20.44
No	1177	21.81
Don’t know	367	6.80
Haven’t heard of low-sodium salt	2749	50.95

*Questions P4 and P5 were skipped if the response to P3 was "Almost none".

Table S3. Associations of HL, eHL, and NSSI with the salt-related knowledge score

	Model 1			Model 2		
	β	95%CI	*P*	*β*	95%CI	*P*
**NSSI**						
0	Ref	-	-	Ref	-	-
1-2	1.039	0.841 to 1.237	<0.001	0.936	0.743 to 1.129	<0.001
3-4	1.754	1.332 to 2.177	<0.001	1.485	1.102 to 1.869	<0.001
5-6	1.745	1.369 to 2.121	<0.001	1.491	1.146 to 1.836	<0.001
7-9	2.157	1.701 to 2.613	<0.001	1.800	1.367 to 2.233	<0.001
**eHL**						
Adequate	0.726	0.477 to 0.976	<0.001	0.296	0.048 to 0.544	0.020
Inadequate	Ref	-	-	Ref	-	-
**HL**						
Adequate	1.388	1.137 to 1.639	<0.001	1.085	0.826 to 1.345	<0.001
Inadequate	Ref	-	-	Ref	-	-

NSSI: number of sources of salt-reduction information; eHL: eHealth literacy; HL: health literacy. Ref: reference group

Model 1 included HL, eHL, and NSSI. Model 2 further adjusted for age, gender, county/district, education, smoking, and physical activity.

Table S4. Associations of HL, eHL, and NSSI with the salt-related attitudes score

	Model 1			Model 2		
	β	95%CI	*P*	*β*	95%CI	*P*
**NSSI**						
0	Ref	-	-	Ref	-	-
1-2	1.168	0.881 to 1.456	<0.001	1.077	0.794 to 1.360	<0.001
3-4	1.417	0.987 to 1.846	<0.001	1.231	0.814 to 1.647	<0.001
5-6	1.807	1.456 to 2.157	<0.001	1.619	1.272 to 1.966	<0.001
7-9	1.712	1.348 to 2.077	<0.001	1.502	1.125 to 1.879	<0.001
**eHL**						
Adequate	0.825	0.593 to 1.058	<0.001	0.649	0.410 to 0.888	<0.001
Inadequate	Ref	-	-	Ref	-	-
**HL**						
Adequate	0.587	0.361 to 0.813	<0.001	0.494	0.259 to 0.729	<0.001
Inadequate	Ref	-	-	Ref	-	-

NSSI: number of sources of salt-reduction information; eHL: eHealth literacy; HL: health literacy. Ref: reference group

Model 1 included HL, eHL, and NSSI. Model 2 further adjusted for age, gender, county/district, education, smoking, and physical activity.

Table S5. Associations of HL, eHL, and NSSI with the salt-related practices score

	Model 1			Model 2		
	β	95%CI	*P*	*β*	95%CI	*P*
**NSSI**						
0	Ref	-	-	Ref	-	-
1-2	0.470	0.292 to 0.647	<0.001	0.395	0.227 to 0.563	<0.001
3-4	0.614	0.391 to 0.836	<0.001	0.513	0.301 to 0.724	<0.001
5-6	0.741	0.547 to 0.936	<0.001	0.626	0.438 to 0.814	<0.001
7-9	0.837	0.565 to 1.109	<0.001	0.748	0.481 to 1.016	<0.001
**eHL**						
Adequate	0.412	0.249 to 0.575	<0.001	0.464	0.300 to 0.628	<0.001
Inadequate	Ref	-	-	Ref	-	-
**HL**						
Adequate	0.143	-0.009 to 0.295	0.065	0.202	0.056 to 0.348	0.008
Inadequate	Ref	-	-	Ref	-	-

NSSI: number of sources of salt-reduction information; eHL: eHealth literacy; HL: health literacy. Ref: reference group

Model 1 included HL, eHL, and NSSI. Model 2 further adjusted for age, gender, county/district, education, smoking, and physical activity.

Table S6. Standardized results of the path model for the salt-related knowledge score

	Estimate	SE	*P*	95%CI	Proportion*(%)
Indirect association 1	0.074	0.011	<0.001	0.053 to 0.097	18.93
Indirect association 2	0.020	0.012	0.083	0.000 to 0.047	5.11
Direct association	0.297	0.041	<0.001	0.219 to 0.377	75.96
Total association	0.391	0.032	<0.001	0.328 to 0.454	-

Indirect association 1: HL → NSSI → Salt-related knowledge.

Indirect association 2: HL → eHL → Salt-related knowledge.

*Refers to the ratio of the specific association to the total association.

Table S7. Standardized results of the path model for the salt-related attitudes score

	Estimate	SE	*P*	95%CI	Proportion*(%)
Indirect association 1	0.070	0.009	<0.001	0.053 to 0.090	31.25
Indirect association 2	0.051	0.015	0.001	0.027 to 0.085	22.77
Direct association	0.103	0.040	0.011	0.027 to 0.187	45.98
Total association	0.224	0.035	<0.001	0.159 to 0.295	-

Indirect association 1: HL → NSSI → Salt-related attitudes.
Indirect association 2: HL → eHL → Salt-related attitudes.
*Refers to the ratio of the specific association to the total association.

Table S8. Standardized results of the path model for the salt-related practices score

	Estimate	SE	*P*	95%CI	Proportion*(%)
Indirect association 1	0.053	0.009	<0.001	0.036 to 0.071	33.76
Indirect association 2	0.057	0.014	<0.001	0.034 to 0.089	36.31
Direct association	0.047	0.033	0.152	-0.018 to 0.110	29.93
Total association	0.157	0.029	<0.001	0.099 to 0.214	-

Indirect association 1: HL → NSSI → Salt-related practices.
Indirect association 2: HL → eHL → Salt-related practices.
*Refers to the ratio of the specific association to the total association.
